# Glycaemic control and complications in haemodialysis patients: the TURK-HEMODIAB Study

**DOI:** 10.1093/ckj/sfaf120

**Published:** 2025-04-18

**Authors:** Ozkan Gungor, Berfu Korucu, Ebru Gok Oguz, Necmi Eren, Zeynep Ural, Hamad Dheir, Erhan Tatar, Ayca Inci, Ismail Kocyigit, Zeki Aydin, Ozkan Ulutas, Ekrem Kara, Orcun Altunoren, Zulfukar Yilmaz, Mustafa Sevinc, Can Sevinc, Atalay Surardamar, Mahmud Islam, Cuneyt Akgol, Halil Zeki Tonbul, Tuba Elif Ozler, Ibrahim Guney, Simal Koksal Cevher, Cihan Uysal, Fatma Betul Guzel, Ruya Mutluay, Zafer Ercan, Semahat Karahisar Sirali, Emre Cankaya, Engin Onan, Ferhan Candan, Eray Eroglu, Neslihan Tezcan, Mehmet Polat, Can Huzmeli, Sultan Ozkurt, Tulin Akagun, Pinar Alp, Ruya Ozelsancak, Gurkan Yurteri, Mehmet Tanrisev, Ali Ilter, Beyhan Guvercin, Mehmet Emin Demir, Ozdem Kavraz Tomar, Alper Azak, Faruk Hilmi Turgut, Mehmet Tuncay, Hakan Akdam, Tamer Selen, Fatih Yilmaz, Ramazan Danis, Sahin Eyupoglu, Gizem Kumru, Simge Bardak Demir, Selma Alagoz, Sami Uzun, Orhan Ozdemir, Zelal Adibelli, Mehmet Polat, Ramazan Ulu, Ahmet Murt, Murside Esra Dolarslan, Ayse Zeynep Bal, Yavuz Ayar, Nazife Nur Ozer Sensoy, Mehmet Sezen, Tuncay Sahutoglu, Ali Veysel Kara, Ercan Turkmen, Hasan Kayabasi, Numan Gorgulu, Ender Hur, Fatma Sibel Kocak Yucel, Zehra Eren, Garip Sahin, Umut Kasapoglu, Zeynep Biyik, Sumeyra Koyuncu, Mehmet Fetullah Aydin, Tolga Yildirim, Gulsah Sasak Kuzgun, Sedat Ustundag, Abdulkadir Celik, Esra Akcali, Ozlem Usalan, Bulent Kaya, Mehmet Riza Altiparmak, Murat Tugcu, Refik Olmaz, Edip Erkus, Zeki Soypacaci, Belma Gokcen Zorba, Demet Yavuz, Kenan Evren Oztop, Melike Betul Ogutmen, Ozlem Harmankaya, Dilek Guven Taymez, Hakan Kaptanogullari, Kemal Magden, Neslihan Seyrek, Haci Bayram Berktas, Mehmet Erdem, Savas Ozturk, Izzet Hakki Arikan, Sim Kutlay, Bulent Altun, Kenan Ates, Mustafa Arici

**Affiliations:** Kahramanmaras Sutcu Imam University, Faculty of Medicine, Department of Nephrology, Kahramanmaras, Turkey; Osmaniye State Hospital, Department of Nephrology, Osmaniye, Turkey; University of Health Sciences, Etlik City Hospital, Department of Nephrology, Ankara, Turkey; Kocaeli University, Faculty of Medicine, Department of Nephrology, Kocaeli, Turkey; Kırıkkale Yüksek Ihtisas Hospital, Department of Nephrology, Kırıkkale, Turkey; Sakarya University Faculty of Medicine, Department of Nephrology, Sakarya, Turkey; Izmir Ekonomi University, Medikalpoint Hospital, Department of Nephrology, Izmir, Turkey; University of Health Sciences, Antalya Training and Research Hospital, Department of Nephrology, Antalya, Turkey; Erciyes University Faculty of Medicine, Department of Nephrology, Kayseri, Turkey; University of Health Sciences, Darica Farabi Training and Research Hospital, Department of Nephrology, Darica, Turkey; Inonu University, Faculty of Medicine, Department of Nephrology, Malatya, Turkey; Recep Tayyip Erdoğan University, Faculty of Medicine, Department of Nephrology, Rize, Turkey; Kahramanmaras Sutcu Imam University, Faculty of Medicine, Department of Nephrology, Kahramanmaras, Turkey; Dicle University, Faculty of Medicine, Department of Nephrology, Diyarbakir, Turkey; Manchester University NHS Trust, Manchester Royal Infirmary, Department of Nephrology, Manchester, UK; Atatürk University, Faculty of Medicine, Department of Nephrology, Erzurum, Turkey; Ministry of Health Beykoz State Hospital, Department of Internal Medicine Beykoz, Istanbul, Turkey; Sakarya University Faculty of Medicine, Department of Nephrology, Sakarya, Turkey; Burdur State Hospital, Department of Nephrology, Burdur, Turkey; Necmettin Erbakan University, Faculty of Medicine, Department of Nephrology, Konya, Turkey; Yeni Yüzyıl University, Gaziosmanpaşa Hospital, Nephrology Department, Istanbul, Turkey; Konya City Hospital, Department of Nephrology, Konya, Turkey; Ministry of Health Ankara Bilkent City Hospital, Department of Nephrology, Ankara, Turkey; Erciyes University Faculty of Medicine, Department of Nephrology, Kayseri, Turkey; Kahramanmaras Sutcu Imam University, Faculty of Medicine, Department of Nephrology, Kahramanmaras, Turkey; Eskişehir Osmangazi University, Faculty of Medicine, Department of Nephrology, Eskişehir, Turkey; Sakarya University Faculty of Medicine, Department of Nephrology, Sakarya, Turkey; Ufuk University Faculty of Medicine, Department of Nephrology, Ankara, Turkey; Ministry of Health Ankara Bilkent City Hospital, Department of Nephrology, Ankara, Turkey; Adana City Hospital, Department of Nephrology, Adana, Turkey; Sivas Cumhuriyet University, Faculty of Medicine, Department of Nephrology, Sivas, Turkey; Kilis State Hospital, Department of Nephrology, Kilis, Turkey; Kütahya University of Health Sciences, Department of Nephrology, Kütahya, Turkey; Nevşehir State Hospital, Department of Nephrology, Nevşehir, Turkey; Hatay Training and Research Hospital, Department of Nephrology, Hatay, Turkey; Eskişehir Osmangazi University, Faculty of Medicine, Department of Nephrology, Eskişehir, Turkey; Giresun University, Faculty of Medicine, Department of Nephrology, Giresun, Turkey; University of Health Sciences, Ankara Training and Research Hospital, Department of Nephrology, Ankara, Turkey; Başkent University, Adana Dr Turgut Noyan Training and Research Hospital, Adana, Turkey; Rentek Dialysis Center, Ankara, Turkey; University of Health Sciences, Tepecik Training and Research Hospital, Department of Nephrology, Izmir, Turkey; Kartal Dr Lutfi Kırdar City Hospital, Department of Nephrology, Istanbul, Turkey; Kanuni Training and Research Hospital, Department of Nephrology, Trabzon, Turkey; Atilim University, Faculty of Medicine, Department of Nephrology, Ankara, Turkey; Giresun University, Faculty of Medicine, Department of Nephrology, Giresun, Turkey; Sağlık Bilimleri University, Balıkesir Atatürk City Hospital, Department of Nephrology, Balıkesir, Turkey; Mustafa Kemal University, Faculty of Medicine, Department of Nephrology, Hatay, Turkey; Dr Ersin Arslan Hospital, Department of Nephrology, Gaziantep, Turkey; Adnan Menderes University, Faculty of Medicine, Department of Nephrology, Aydın, Turkey; Düzce Atatürk State Hospital, Department of Nephrology, Düzce, Turkey; Antalya Atatürk State Hospital, Department of Nephrology, Antalya, Turkey; Gazi Yasargil Training and Research Hospital, Department of Nephrology, Diyarbakır, Turkey; Ağrı State Hospital, Department of Nephrology, Ağrı, Turkey; Ankara University, Faculty of Medicine, Department of Nephrology, Ankara, Turkey; Yenimahalle Training and Research Hospital, Department of Nephrology, Ankara, Turkey; Bağcılar Training and Research Hospital, Department of Nephrology, Istanbul, Turkey; Haseki Training and Research Hospital, Department of Nephrology, Istanbul, Turkey; Şanlıurfa Training and Research Hospital, Department of Nephrology, Şanlıurfa, Turkey; Usak University, Faculty of Medicine, Department of Nephrology, Usak, Turkey; Karabuk Training and Research Hospital, Department of Nephrology, Karabük, Turkey; Adıyaman University, Faculty of Medicine, Department of Nephrology, Adıyaman, Turkey; Bingöl State Hospital, Department of Nephrology, Bingöl, Turkey; Mersin City Training and Research Hospital, Department of Nephrology, Mersin, Turkey; University of Health Sciences, Ankara Training and Research Hospital, Department of Nephrology, Ankara, Turkey; University of Health Sciences, Bursa Faculty of Medicine, Bursa City Hospital, Bursa, Turkey; University of Health Sciences, Bursa Faculty of Medicine, Bursa City Hospital, Bursa, Turkey; University of Health Sciences, Bursa Faculty of Medicine, Bursa City Hospital, Bursa, Turkey; Mehmet Akif Inan Training and Research Hospital, Department of Nephrology, Şanlıurfa, Turkey; Erzincan Binali Yıldırım University, Faculty of Medicine, Department of Nephrology, Erzincan, Turkey; Ondokuz Mayıs University, Faculty of Medicine, Department of Nephrology, Samsun, Turkey; University of Health Sciences, Umraniye Training and Research Hospital, Department of Nephrology, Istanbul, Turkey; Bağcılar Training and Research Hospital, Department of Nephrology, Istanbul, Turkey; Usak University, Faculty of Medicine, Department of Nephrology, Usak, Turkey; University of Health Sciences, Istanbul Bakirköy Sadi Konuk Education and Research Hospital, Istanbul, Turkey; Alaaddin Keykubat University, Department of Nephrology, Antalya, Turkey; Eskişehir Osmangazi University, Faculty of Medicine, Department of Nephrology, Eskişehir, Turkey; University of Health Sciences, Istanbul Bakirköy Sadi Konuk Education and Research Hospital, Istanbul, Turkey; Selcuk University Faculty of Medicine, Deparment of Nephrology, Konya, Turkey; Kayseri City Hospital, Department of Nephrology, Kayseri, Turkey; Sağlık Bilimleri University, Balıkesir Atatürk City Hospital, Department of Nephrology, Balıkesir, Turkey; Hacettepe University, Faculty of Medicine, Department of Nephrology, Ankara, Turkey; Prof. Dr Süleyman Yalçın City Hospital, Department of Nephrology, Istanbul, Turkey; Trakya University, Faculty of Medicine, Department of Nephrology, Edirne, Turkey; Haseki Training and Research Hospital, Department of Nephrology, Istanbul, Turkey; Tarsus State Hospital, Department of Nephrology, Mersin, Turkey; Gaziantep University, Faculty of Medicine, Department of Nephrology, Gaziantep, Turkey; Çukurova University, Faculty of Medicine, Department of Nephrology, Adana, Turkey; Istanbul University-Cerrahpaşa, Cerrahpaşa Faculty of Medicine, Department of Nephrology, Istanbul, Turkey; Marmara University, Faculty of Medicine, Department of Nephrology, Istanbul, Turkey; Mersin City Training and Research Hospital, Department of Nephrology, Mersin, Turkey; Erzurum City Hospital, Department of Nephrology, Erzurum, Turkey; Izmir Katip Celebi University, Atatürk Training and Research Hospital, Department of Nephrology, Izmir, Turkey; Manisa Merkezefendi State Hospital, Department of Nephrology, Manisa, Turkey; Samsun University, Faculty of Medicine, Department of Nephrology, Samsun, Turkey; Sakarya University Faculty of Medicine, Department of Nephrology, Sakarya, Turkey; University of Health Sciences, Haydarpaşa Numune Education and Research Hospital, Department of Nephrology, Istanbul, Turkey; Biruni University, Faculty of Health Sciences, Department of Nephrology, Istanbul, Turkey; Kocaeli City Hospital, Department of Nephrology, Kocaeli, Turkey; Biruni University, Faculty of Health Sciences, Department of Nephrology, Istanbul, Turkey; Gebze Fatih State Hospital, Department of Nephrology, Kocaeli, Turkey; Çukurova University, Faculty of Medicine, Department of Nephrology, Adana, Turkey; Inonu University, Faculty of Medicine, Department of Nephrology, Malatya, Turkey; Yüzüncü Yıl University, Faculty of Medicine, Department of Internal Medicine, Van, Turkey; Istanbul University, Istanbul Faculty of Medicine, Department of Internal Medicine, Division of Nephrology, Istanbul, Türkiye; Marmara University, Faculty of Medicine, Department of Nephrology, Istanbul, Turkey; Ankara University, Faculty of Medicine, Department of Nephrology, Ankara, Turkey; Hacettepe University, Faculty of Medicine, Department of Nephrology, Ankara, Turkey; Ankara University, Faculty of Medicine, Department of Nephrology, Ankara, Turkey; Hacettepe University, Faculty of Medicine, Department of Nephrology, Ankara, Turkey

**Keywords:** complication, diabetic, glycaemic control, HbA1c, haemodialysis

## Abstract

**Background:**

The most common cause of end-stage kidney disease is diabetes mellitus (DM). The most commonly used renal replacement therapy in Turkey and in many countries around the world is haemodialysis (HD). Glycaemia control is important in these populations. In this study we aimed to screen for glycaemic control and complications in a large population of diabetic HD patients in Turkey.

**Methods:**

A total of 16 043 patients were screened in 253 dialysis centres in Turkey and 5038 diabetic HD patients were included in the study. At participating centres, patients’ diabetes history, complications, medications, haemoglobin A1c (HbA1c) and other laboratory data were reviewed and recorded by nephrologists.

**Results:**

The average age of the patients was 64.0 ± 11.2 years and 56% were male. The mean HbA1c was 7.4 ± 1.5%. Patients were divided into three groups according to the HbA1c level (<6.5%, 6.5–8% and >8%). As the HbA1c levels increased, the mean systolic blood pressure and diastolic blood pressure increased significantly. In addition, as the HbA1c levels increased, the number of patients with coronary artery disease, patients undergoing coronary artery bypass graft surgery and the rate of patients with diabetic retinopathy and vision loss increased. Diabetic foot disease and amputation rates were also higher in the group with poor glycaemic control. The number of patients using intensive or mixed insulin was also higher in the group with high HbA1c levels. In ordinal logistic regression analysis, age significantly decreased and higher body mass index slightly increased the risk of a higher HbA1c. Also, the need for a diabetic diet was greater in those with high HbA1c levels.

**Conclusion:**

Our study highlights that the target values for diabetic HD patients in Turkey are partially compatible with the 2022 Kidney Disease: Improving Global Outcomes guidelines for diabetes management. Nevertheless, more effort and teamwork are needed to improve patient outcomes.

KEY LEARNING POINTS **What was known:**

- Diabetic patients on haemodialysis (HD) face a higher risk of severe complications, including cardiovascular disease, due to the compounded effects of chronic kidney disease (CKD) and poorly controlled blood glucose levels.
- There has been limited data on the demographic and clinical characteristics of diabetic HD patients in Turkey, with little focus on how haemoglobin A1c (HbA1c) levels relate to health outcomes in this specific population

**This study adds:**

- This study demonstrates a clear association between higher HbA1c levels and an increased prevalence of both cardiovascular and diabetes-related complications in diabetic HD patients, highlighting the potential of HbA1c as a valuable risk stratification marker in this population.

**Potential impact:**

- The study emphasizes the necessity of involving multidisciplinary teams, including nephrologists, endocrinologists and dietitians, to create comprehensive, patient-specific glycaemic management plans for diabetic HD patients, ensuring better health outcomes.
- Insights from this study could encourage healthcare providers to implement closer and more proactive monitoring of HbA1c levels in diabetic HD patients, facilitating early intervention for those at higher risk of complications and potentially lowering healthcare costs associated with complex diabetes care.
- Improved glycaemic control through personalized care strategies can reduce the burden of cardiovascular complications and enhance the overall health of diabetic HD patients.

## INTRODUCTION

As the number of individuals with diabetes mellitus (DM) is rapidly increasing worldwide, the prevalence of chronic kidney disease (CKD) among people with diabetes is also increasing [1]. In Turkey, according to epidemiological studies [Turkish Diabetes Epidemiology Study I and II (TURDEP I and TURDEP II)], the frequency of diabetes is between 7.2% and 13.7% [2, 3]. The risk of developing CKD doubles in individuals with diabetes and the incidence of CKD is reported to range from 27.1% to 83.6%, depending on the geographical location [4].

Globally, diabetes is the most common aetiology for end-stage kidney disease (ESKD). According to the Turkish Nephrology Association's 2022 Turkish Kidney Registry System report, the rate of diabetes among incident patients diagnosed with ESKD and starting renal replacement therapy was 36.7%. Among haemodialysis (HD) patients, this rate was found to be highest at 38.4% [5]. The two most significant factors negatively affecting prognosis in dialysis patients are advanced age and the presence of other chronic diseases, especially diabetes [6].

A crucial distinction between diabetic and non-diabetic HD patients is the significantly lower survival rate in diabetic HD patients. The leading cause of mortality in diabetic HD patients is cardiovascular diseases, with a 2–5 times higher risk compared with non-diabetic dialysis patients [7].

Thus a holistic and multidisciplinary approach is essential in treating diabetic kidney disease in HD. Physicians dealing with dialysis should be aware of the challenges posed by diabetic patients and be prepared to manage the complications. The interventions should focus on achieving metabolic control, and patients should also be educated and included in the management of their disease and complications. In disease management, ensuring regular prescribed medication use, self-monitoring of blood glucose and active participation in preventing diabetes complications should be emphasized.

In monitoring metabolic control among diabetic patients, haemoglobin A1c (HbA1c) remains the most widely utilized and reliable indicator. Elevated HbA1c levels correlate with the development of both microvascular and macrovascular complications [8].

The number of diabetic HD patients in Turkey is high and this number is increasing. However, we do not have data on the glycaemic control and complications of these patients. Our study aims to comprehensively assess the disease management status of diabetic HD patients in seven regions of Turkey. We focused on evaluating glycaemic control measures and conducting a thorough screening for complications. Given the higher mortality and morbidity rates in diabetic HD patients compared with other dialysis patients, our research aims to provide insights into optimizing the care and outcomes of this specific patient population.

## MATERIALS AND METHODS

### Study population and data collection

**Figure 1:**
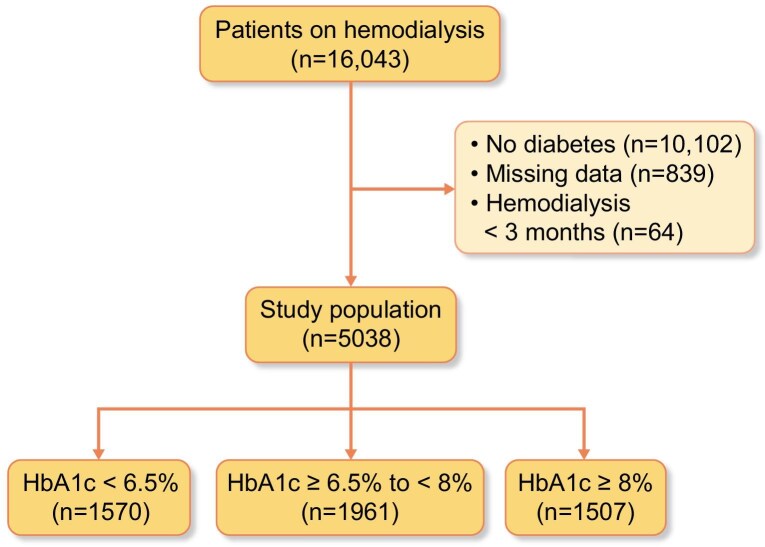
Study flow chart of diabetic HD patients. Flow diagram illustrating the selection process of HD patients with diabetes from initial screening to inclusion in the study, categorized by HbA1c levels.

In this study, 16 043 patients were screened for treatment in 253 dialysis centres in seven regions across Turkey. A total of 5038 (31%) diabetic HD patients ≥18 years of age and receiving HD treatment for ≥3 months were included. Ethical approval was obtained from the Institutional Review Board of Istanbul Bakirkoy Sadi Konuk Training and Research Hospital (approval no. 2021/558) and written informed consent was obtained from all participating patients. The study adhered to the principles of the Helsinki Declaration. The study flow chart is shown in Fig. 1.

Nephrology specialists at the participating centres collected data from diabetic HD patients under their care, reviewing medical records and examining laboratory data. Patient demographics, including age, gender, education level, monthly income, smoking status, causes of CKD, duration of HD treatment, transplantation history, hepatitis serology panels, active vascular access and vascular access intervention history, duration of diabetes, additional comorbidities, diabetic retinopathy, symptomatic hypoglycaemia (defined as the patient having symptoms of hypoglycaemia and a blood glucose level <50 mg/dl) and the number of symptomatic hypoglycaemia episodes requiring treatment at any time after starting dialysis, amputation history, diabetic treatments, antihypertensive medications and other medications, were recorded. Laboratory data were taken from the patients’ examinations performed in the last month. Daugirdas’s second-generation equation was used to calculate urea clearance (*Kt*/*V*) and the urea reduction ratio (URR) was calculated by dividing the difference between pre- and post-dialysis urea levels by pre-dialysis urea levels. HbA1c was measured nationwide using high-performance liquid chromatography.

The Kidney Disease: Improving Global Outcomes (KDIGO) 2022 diabetic kidney disease guidelines divide CKD patients into three groups according to the last measured HbA1c value (<6.5%, 6.5–8% and >8%), thus patients were divided into three groups. The groups’ baseline characteristics, laboratory values, primary kidney diseases, comorbidities, blood pressures (BPs), HD prescriptions, measures regarding HD adequacy and vascular access routes were compared. To understand the diabetic burden of the patients, the diabetic complications, medications used and relevant laboratory data of the groups were compared. The risks posed by the increasing HbA1c values of the groups were examined.

### Statistical methods

The numeric values are shown as means and standard deviations (SDs) or medians with interquartile ranges (IQRs) according to the distributions. Categorical data are shown as frequencies and percentages. We compared the categorical variables with the chi-squared test where applicable. We used the analysis of variance (ANOVA) test to compare normally distributed data and the Kruskal–Wallis test for non-normally distributed data. For the post hoc analyses, we used the ANOVA test with Bonferroni correction. We conducted ordinal logistics regression analysis to evaluate risk regarding involvement in increasing HbA1c categories. The analyses considered a *P*-value <.05 (5% of type I error level) as statistically significant. All statistical analyses were performed using the Statistical Package for Social Science, version 26.0 (IBM, Armonk, NY, USA).

## RESULTS

### Baseline characteristics of the groups

All demographic and laboratory characteristics of the study population are shown in Table 1. The distribution of sex, education status, frequency of smoking and mean income were similar between the groups according to HbA1c levels. Patients in the HbA1c >8% group were significantly younger and had a higher mean body mass index (BMI) compared with the study population (*P* < .01 for both). A total of 855 (16.97%) patients had HbA1c levels <5% and 241 (4.78%) patients had HbA1c levels >10%. The causes of primary kidney disease showed a significant difference between groups, as shown in Table 1 (*P* < .01). The percentage of patients with renal transplantation before HD and mean HD vintage were similar between groups. As the HbA1c level of the groups increased, the rate of hypertensive patients increased significantly (*P* = .03).

**Table 1:** Demographic and laboratory characteristics of the study population.

Characteristics	Study population (*n* = 5038)	HbA1c <6.5% (*n* = 1570)	HbA1c ≥6.5—<8% (*n* = 1961)	HbA1c ≥8% (*n* = 1507)	*P*-value
Age (years), mean ± SD	64.0 ± 11.2	65.8 ± 11.2	64.5 ± 10.7	61.4 ± 11.5	**<.01**
Female, *n* (%)	2215 (44)	710 (45.2)	856 (43.7)	649 (43.1)	.45
BMI (kg/m^2^), mean ± SD	27.6 ± 5.5	27.0 ± 5.2	27.8 ± 5.7	27.9 ± 5.6	**<.01**
Primary kidney disease, *n* (%)	Unknown: 229 (4.5)	Unknown: 96 (6.1)	Unknown: 83 (4.2)	Unknown: 50 (3.3)	**<.01**
	DM: 4199 (83.3)	DM: 1216 (77.5)	DM: 1658 (84.5)	DM: 1325 (87.9)	
	HT: 313 (6.2)	HT: 121 (7.7)	HT: 113 (5.8)	HT: 79 (5.2)	
	GN/Vasc: 62 (1.2)	GN/Vasc: 32 (2.0)	GN/Vasc: 19 (1.0)	GN/Vasc: 11 (0.7)	
	ADPKD: 53 (1.1)	ADPKD: 21 (1.3)	ADPKD: 23 (1.2)	ADPKD: 9 (0.6)	
	Amyloidosis: 6 (0.1)	Amyloidosis: 4 (0.3)	Amyloidosis: 0 (0.0)	Amyloidosis: 2 (0.1)	
	Urologic: 65 (1.3)	Urologic: 26 (1.7)	Urologic: 26 (1.3)	Urologic: 13 (0.9)	
	Other: 111 (2.2)	Other: 54 (3.4)	Other: 39 (2.0)	Other: 18 (1.2)	
Transplantation, *n* (%)	132 (2.6)	53 (3.4)	46 (2.3)	34 (2.3)	.15
Hypertension, *n* (%)	4175 (82.9)	1278 (81.2)	1622 (82.7)	1278 (84.8)	**.03**
HD vintage (days), median (IQR)	36 (15–70)	36 (15–70)	36 (15–66)	36 (17–72)	.39
Vascular access, *n* (%)	AVF: 3564 (70.7)	AVF: 1086 (69.2)	AVF: 1392 (71.0)	AVF: 1086 (72.1)	.18
	AVG: 48 (1.0)	AVG: 12 (0.8)	AVG: 25 (1.3)	AVG: 11 (0.7)	
	Temp cath: 94 (1.9)	Temp cath: 30 (1.9)	Temp cath: 34 (1.7)	Temp cath: 30 (2.0)	
	Tunnelled cath: 1332 (26.4)	Tunnelled cath: 442 (28.2)	Tunnelled cath: 510 (26.0)	Tunnelled cath: 380 (25.2)	
Vascular access time (months), median (IQR)	24 (9–48)	24 (9–48)	24 (10–48)	22 (9–48)	.65
Total AVF/AVG operations, median (IQR)	1 (1–2)	1 (1–2)	1 (1–2)	1 (1–2)	**<.01**
HD sessions per week, median (IQR)	3 (3–3)	3 (3–3)	3 (3–3)	3 (3–3)	**<.01**
HD timing, *n* (%)	Morning: 2289 (45.4)	Morning: 706 (45)	Morning: 896 (45.7)	Morning: 687 (45.6)	.91
	Noon: 2294 (45.5)	Noon: 723 (46.1)	Noon: 892 (45.5)	Noon: 679 (45.1)	
	Evening: 455 (9.0)	Evening: 141 (9.0)	Evening: 173 (8.8)	Evening: 141 (9.4)	
SBP (mmHg), mean ± SD	132.8 ± 19.3	130.9 ± 19.3	132.8 ± 18.7	134.9 ± 19.9	**<.01**
DBP (mmHg), mean ± SD	76.9 ± 11.0	76.2 ± 11.2	76.7 ± 10.6	77.6 ± 11.3	**<.01**
HD duration (minutes), mean ± SD	240.2 ± 8.5	240.2 ± 9.6	240.2 ± 9.5	240.1 ± 8.5	.54
UF (l/day), mean ± SD	2.8 ± 0.9	2.7 ± 1.0	2.9 ± 1.0	3.0 ± 1.0	**<.01**
Kt/V, mean ± SD	1.54 ± 0.3	1.57 ± 0.3	1.53 ± 0.3	1.52 ± 0.3	**<.01**
URR, mean ± SD	71.9 ± 7.1	72.4 ± 6.9	71.7 ± 7.2	71.8 ± 7.0	.36
RRF (ml/day), median (IQR)	100 (0–500)	100 (0–500)	100 (0–400)	100 (0–450)	.32

GN/Vasc: glomerulonephritis/vascular disease; ADPKD: autosomal dominant polycystic kidney disease; Temp cath: temporary catheter: Tunnelled cath: tunnelled catheter; RRF: residual renal function.

Significant values in bold.

The vascular access type distributions of the groups were similar. As the HbA1c level of the groups increased, the number of arteriovenous fistula (AVF)/arteriovenous graft (AVG) operations and the weekly HD sessions increased slightly (*P* < .01 for both). The mean systolic blood pressure (SBP) and diastolic blood pressure (DBP) of the groups increased significantly as the HbA1c levels of the groups increased (*P* < .01 for both). The groups’ mean ultrafiltration (UF) amount per day significantly increased and the mean Kt/V slightly decreased as the groups’ HbA1c levels increased (*P* < .01 for both).

### Comorbid diseases of the groups

The frequencies of patients with hepatitis B virus (HBV) and hepatitis C virus (HCV) were similar between the groups. The frequencies of coronary artery disease (CAD), patients with one or more coronary stents and patients who underwent coronary artery bypass graft (CABG) surgery significantly increased as the groups’ HbA1c levels increased (*P* < .01 for all). Patients with documented congestive heart failure (CHF) were similar among the groups, but the number of patients with an ejection fraction (EF) of <40% increased as the groups’ HbA1c levels increased (*P* = .08 and *P* = .01, respectively). The number of patients with cardiovascular disease (CVD) was significantly different, with the highest number in the HbA1c ≥6.5–8% group (*P* = .02) (Table 2).

**Table 2:** Comorbid diseases of the groups.

Comorbid diseases	Study population (*n* = 5038)	HbA1c <6.5% (*n* = 1570)	HbA1c ≥6.5–<8% (*n* = 1961)	HbA1c ≥8% (*n* = 1507)	*P*-value
CAD, *n* (%)	1979 (39.3)	557 (35.5)	735 (37.5)	687 (45.6)	**<.01**
Coronary stent, *n* (%)	1060 (21.0)	266 (16.9)	413 (21.1)	381 (25.3)	**<.01**
CABG, *n* (%)	613 (12.2)	160 (10.2)	230 (11.7)	223 (14.8)	**<.01**
CHF, *n* (%)	1070 (21.2)	321 (20.4)	399 (20.3)	350 (23.2)	.08
EF <40%, *n* (%)	332 (6.6)	87 (5.5)	120 (6.1)	125 (8.3)	**.01**
Cerebrovascular disease, *n* (%)	409 (8.1)	109 (6.9)	185 (9.4)	115 (7.6)	**.02**
Malignant disease, *n* (%)	178 (3.5)	74 (4.7)	62 (3.2)	42 (2.8)	**.01**
CLD, *n* (%)	95 (1.9)	30 (1.9)	33 (1.7)	32 (2.1)	.64
Autoimmune disease, n (%)	149 (3.0)	47 (3.0)	55 (2.8)	47 (3.1)	.86
COPD, *n* (%)	638 (12.7)	185 (11.8)	240 (12.2)	213 (14.1)	.11
Blood transfusion within 6 months, *n* (%)	495 (9.8)	164 (10.4)	189 (9.6)	142 (9.4)	.61

CLD: chronic liver disease; COPD: chronic obstructive pulmonary disease.

Significant values in bold.

### DM type and related conditions and complications of the groups

The number of patients with diabetic retinopathy (DRP) significantly increased and higher vision loss stages were observed as the groups’ HbA1c levels increased (*P* < .01 for both). The number of patients with diabetic foot and amputation rates increased significantly as the groups’ HbA1c levels increased (*P* = .03 and *P* < .01, respectively).

The number of patients using a glucometer, who perform routine home glucose measurements and who experienced one or more symptomatic hypoglycaemia episode and hypoglycaemic coma significantly increased as the groups’ HbA1c levels increased (*P* < .01 for all). More patients in the group with HbA1c >8% had a professional diabetic diet (*P* = .03). The medical doctors or specialists preferred by the groups for DM follow-up visits were significantly different, as described in Table 3 (*P* = .04).

**Table 3:** DM type and related conditions and complications of the groups.

Conditions/complications	Study population (*n* = 5038)	HbA1c <6.5% (*n* = 1570)	HbA1c ≥6.5–<8% (*n* = 1961)	HbA1c ≥8% (*n* = 1507)	*P*-value
DM type, *n* (%)	Type 1: 414 (8.2)	Type 1: 100 (6.4)	Type 1: 149 (7.6)	Type 1: 165 (10.9)	**<.01**
	Type 2: 4615 (91.6)	Type 2: 1467 (93.4)	Type 2: 1089 (92.2)	Type 2: 1339 (88.9)	
	MODY: 9 (0.2)	MODY: 3 (0.2)	MODY: 3 (0.2)	MODY: 3 (0.2)	
DM vintage years, median (IQR)	18 (10–24)	15 (10–22)	18 (10–25)	20 (14–25)	**<.01**
DRP, *n* (%)	No: 1730 (34.3)	No: 676 (43.0)	No: 660 (33.7)	No: 394 (26.1)	**<.01**
	Yes: 2604 (51.7)	Yes: 659 (42.0)	Yes: 1039 (53.0)	Yes: 906 (60.1)	
	Unknown: 704 (14.0)	Unknown: 235 (15.0)	Unknown: 262 (13.3)	Unknown: 207 (13.8)	
DRP time (years), median (IQR)	6 (3–10)	6 (3–10)	6 (3–10)	6 (4–10)	**.03**
Vision loss, *n* (%)	None: 1688 (33.5)	None: 635 (40.4)	None: 642 (32.7)	None: 411 (27.3)	**<.01**
	Decreased: 3190 (63.3)	Decreased: 892 (56.8)	Decreased: 1251 (63.8)	Decreased: 1047 (69.5)	
	Blindness: 148 (2.9)	Blindness: 42 (2.7)	Blindness: 59 (3.0)	Blindness: 47 (3.1)	
	Unknown: 12 (0.2)	Unknown: 1 (0.1)	Unknown: 9 (0.5)	Unknown: 2 (0.1)	
Diabetic foot, *n* (%)	904 (17.9)	234 (14.9)	340 (17.3)	330 (21.9)	**<.01**
Amputation, *n* (%)	378 (7.5)	99 (6.3)	146 (7.4)	133 (8.8)	**.03**
Glucometer user, *n* (%)	4523 (89.8)	1326 (84.5)	1787 (91.1)	1410 (93.6)	**<.01**
Home glucose measurement, *n* (%)	3820 (75.8)	1084 (69.0)	1521 (77.6)	1215 (80.6)	**<.01**
Symptomatic hypoglycaemia, *n* (%)	1925 (38.2)	464 (29.6)	751 (38.3)	710 (47.1)	**<.01**
Emergency admission due to hypoglycaemia, *n* (%)	724 (14.4)	148 (9.4)	264 (13.5)	312 (20.7)	**<.01**
Diabetic diet, *n* (%)	2677 (53.1)	827 (52.7)	1031 (52.6)	819 (54.3)	**.03**
DM follow-up, *n* (%)	Self-reg: 381 (7.6)	Self-reg: 127 (8.1)	Self-reg: 139 (7.1)	Self-reg: 115 (7.6)	**.04**
	Primary care: 65 (1.3)	Primary care: 22 (1.4)	Primary care: 28 (1.4)	Primary care: 15 (1.0)	
	HD pract: 572 (11.4)	HD pract: 193 (12.3)	HD pract: 216 (11.0)	HD pract: 163 (10.8)	
	Internist: 585 (11.6)	Internist: 166 (10.6)	Internist: 236 (12.0)	Internist: 183 (12.1)	
	Endocrinologist: 122 (2.4)	Endocrinologist: 29 (1.8)	Endocrinologist: 54 (2.8)	Endocrinologist: 39 (2.6)	
	Nephrologist: 137 (2.7)	Nephrologist: 35 (2.2)	Nephrologist: 43 (2.2)	Nephrologist: 59 (3.9)	
	Other 3176 (63.0)	Other 998 (63.6)	Other 1245 (63.5)	Other 933 (61.9)	

MODY: maturity onset diabetes of the young; Sel-reg: self-regulation; HD pract: haemodialysis practitioner,

Other: any physician the patient can reach at that moment (HD practitioner, internist, endocrinologist, or nephrologist).

Significant values in bold.

### Medical treatments of the groups

The groups’ medical treatments related to DM, hypertension and CKD are shown in Table 4. The number of the patients using intensive (basal insulin + bolus insulin combined administration) or mixed insulin increased as the groups’ HbA1c levels increased (*P* < .01 for both). More patients were using single-dose insulin in the HbA1c ≥6.5–8% group (*P* < .01). Oral antidiabetic drug (OAD) use frequencies were similar among the groups, except for the significantly lower use of thiazolidinediones in the HbA1c >8% group (*P* = .04). Combined OAD use and the number patients without the need for medications for DM significantly decreased and any antidiabetic use on HD day increased significantly as the groups’ HbA1c levels increased (*P* = .01, *P* < .01 and *P* < .01, respectively).

**Table 4:** Medical treatments of the groups.

Treatments	Study population (*n* = 5038)	HbA1c <6.5% (*n* = 1570)	HbA1c ≥6.5–<8% (*n* = 1961)	HbA1c ≥8% (*n* = 1507)	*P*-value
Intensive insulin, *n* (%)	1458 (28.9)	301 (19.2)	516 (26.3)	641 (42.5)	**<0.01**
Mixed insulin, *n* (%)	1104 (21.9)	221 (14.1)	470 (24.0)	413 (27.4)	**<0.01**
Single-dose insulin, *n* (%)	1044 (20.7)	288 (18.3)	463 (23.6)	293 (19.4)	**<0.01**
Metformin, *n* (%)	107 (2.1)	31 (2.0)	52 (2.7)	24 (1.6)	0.09
Sulfonylurea, *n* (%)	77 (1.5)	19 (1.2)	38 (1.9)	20 (1.3)	0.16
Thiazolidinedione, *n* (%)	46 (0.9)	17 (1.1)	23 (1.2)	6 (0.4)	**0.04**
DPP4 inhibitors, *n* (%)	560 (11.1)	181 (11.5)	226 (11.5)	153 (10.2)	0.37
Combined OADs, *n* (%)	305 (6.1)	106 (6.8)	131 (6.7)	68 (4.5)	**0.01**
No meds for DM, *n* (%)	667 (13.2)	409 (26.1)	195 (9.9)	63 (4.2)	**<0.01**
Antidiabetics on HD day, *n* (%)	985 (19.6)	262 (16.7)	377 (19.2)	346 (23.0)	**<0.01**
ACEIs, *n* (%)	759 (15.1)	233 (14.8)	301 (15.3)	225 (14.9)	0.09
ARBs, *n* (%)	391 (7.8)	85 (5.4)	177 (9.0)	129 (8.6)	**<0.01**
Calcium channel blockers, *n* (%)	2156 (42.8)	660 (42.0)	813 (41.5)	683 (45.3)	0.06
Beta blockers, *n* (%)	2448 (48.6)	732 (46.6)	943 (48.1)	773 (51.3)	**0.03**
Statins, *n* (%)	1120 (22.2)	229 (19.0)	424 (21.6)	397 (26.3)	**<0.01**
Fibrates, *n* (%)	142 (2.8)	42 (2.7)	46 (2.3)	54 (3.6)	0.09
Acetylsalicylic acid, *n* (%)	3401 (67.5)	1049 (66.8)	1297 (66.1)	1055 (70.0)	**0.04**
Anticoagulants, *n* (%)	1609 (31.9)	499 (31.8)	601 (30.6)	509 (33.8)	0.15
Calcium-based phosphate binders, *n* (%)	2921 (58.0)	906 (57.7)	1096 (55.9)	919 (61.0)	**0.01**
Sevelamer, *n* (%)	1229 (24.4)	391 (24.9)	483 (24.6)	355 (23.6)	0.65
Active vitamin D and analogues, *n* (%)	2543 (50.5)	755 (48.1)	1009 (51.5)	779 (51.7)	**0.07**
Potassium binders, *n* (%)	831 (16.5)	238 (15.2)	322 (16.4)	271 (18.0)	0.11
Intravenous iron, *n* (%)	3544 (70.3)	1111 (70.8)	1368 (69.8)	1065 (70.7)	0.77
ESA, *n* (%)	3722 (73.9)	1199 (76.4)	1403 (71.5)	1120 (74.3)	**0.01**
ESA dose (U/kg/week), median (IQR)	78 (0–144)	85 (25–150)	75 (0–140)	75 (0–146.5)	**<0.01**
Glucocorticoids, *n* (%)	74 (1.5)	29 (1.8)	24 (1.2)	21 (1.4)	0.29

Significant values in bold.

The frequency of angiotensin-converting enzyme inhibitor (ACEI) use was similar and there was a significantly lower number of patients using angiotensin receptor blockers (ARBs) in the HbA1c <6.5% group (*P* = .09 and *P* < .01, respectively). The frequency of statin use increased significantly as the groups’ HbA1c levels and fibrate use were similar (*P* < .01 and *P* = .09, respectively). The HbA1c >8% group had the highest calcium-based phosphate binder use frequency (*P* = .01). Sevelamer, active vitamin D or active D vitamin D analogues, potassium binders and intravenous iron use frequencies were similar between the groups. The HbA1c ≤6.5% group had the highest erythropoietin-stimulating agents (ESAs) use frequency and weekly ESA dose (*P* = .01 and *P* < .01, respectively). Glucocorticoid use was similar among the groups.

### Laboratory parameters

The mean morning fasting glucose levels increased significantly as the groups’ HbA1c levels increased (*P* < .01). The mean haemoglobin and albumin levels were similar among the groups (*P* = .12 and *P* = .37, respectively). Detailed laboratory parameters are presented in Table 5.

**Table 5:** Laboratory parameters of the groups.

Parameters	Study population (*n* = 5038)	HbA1c <6% (*n* = 1570)	HbA1c ≥6–<8 (*n* = 1961)	HbA1c ≥8% (*n* = 1507)	*P*-value
Glucose (mg/dl), mean ± SD	182.2 ± 79.6	141.3 ± 52.5	176.1 ± 63.4	232.6 ± 93.5	**<.01**
HbA1c (%), mean ± SD	7.4 ± 1.5	5.8 ± 0.4	7.2 ± 0.4	9.2 ± 1.1	**<.01**
Haemoglobin (g/dl), mean ± SD	10.8 ± 1.5	10.7 ± 1.4	10.9 ± 1.5	10.8 ± 1.4	.12
Albumin (g/dl), mean ± SD	3.8 ± 0.4	3.8 ± 0.4	3.7 ± 0.4	3.7 ± 0.3	.37

### Ordinal logistic regression analysis according to HbA1c groups

Age significantly decreases and higher BMI slightly increases the risk of higher HbA1c {odds ratio [OR] 0.97 [95% confidence interval (CI) 0.96–0.98], *P* < .01 and OR 1.02 [95% CI 1.01–1.03], *P* < .01, respectively}. The risk of a higher HbA1c level slightly increases with DM vintage [OR 1.02 (95% CI 1.01–1.02), *P* < .01]. Patients with a professional DM diet tend to have higher HbA1c levels [OR 0.63 (95% CI 0.43–0.92), *P* = .02]. Higher fasting glucose levels are slightly related to the risk of higher HbA1c levels [OR 1.01 (95% CI 1.01–1.02), *P* = .03]. Gender, HD vintage, cardiovascular comorbidities, diabetic foot, routine home glucose measurements, symptomatic hypoglycaemia episodes, weekly ESA dose and haemoglobin levels do not predict HbA1c levels. A summary of the ordinal logistic regression results is presented in Table 6.

**Table 6:** Ordinal logistic regression analysis according to HbA1c groups.

	OR (95% CI)	*P*-value
Age (years), mean	0.97 (0.96–0.98)	**<.01**
Female (n)	1.13 (0.98–1.30)	.09
BMI, mean	1.02 (1.01–1.03)	**<.01**
HD time (months), median	0.99 (0.99–1.00)	.63
DM time (years)	1.02 (1.01–1.02)	**<.01**
CAD (n)	0.87 (0.76–1.02)	.08
CHF (n)	0.85 (0.71–1.01)	.06
CVD (n)	0.90 (0.71–1.15)	.41
Diabetic foot (n)	0.91 (0.76–1.09)	.33
Home glucose measurement (n)	1.23 (0.75–2.02)	.41
Diabetic diet (n)	0.63 (0.43–0.92)	**.02**
Symptomatic hypoglycaemia (n)	1.02 (0.74–1.43)	.89
Weekly ESA dose, mean	0.99 (0.99–1.00)	.80
Glucose, mean	1.01 (1.01–1.02)	**<.01**
Haemoglobin, mean	1.03 (0.98–1.09)	.27

Significant values in bold.

## DISCUSSION

Our study is the most extensive dataset on diabetic HD patients available in the literature, offering crucial insights into the intricate relationship between glycaemic control and various diabetes-related complications.

However, establishing the benefits of tight glycaemic control in diabetic patients undergoing HD remains challenging, with a need for caution to prevent hypoglycaemia, a potentially life-threatening complication. The HbA1c values considered optimal for the average diabetic population, i.e. <7%, prove unrealistic for HD patients. The 2022 KDIGO guidelines for diabetic kidney diseases recommend a target HbA1c of <8% (<64 mmol/mol) for HD patients [9]. While the target HbA1c for diabetic CKD falls within the range of 6.5–8%, there is no validated optimal range for patients on dialysis, with a value close to 8% being suggested.

In our study, ≈38% of our patients had HbA1c measurements between 6.5–8%, suggesting a reasonably controlled diabetes status. However, a concerning finding was that nearly 30% of the patients had HbA1c levels >8%, indicating inadequate overall diabetes regulation. Notably, HbA1c values <6% may raise concerns about malnutrition [9]. Conversely, HbA1c values >10% in HD patients are definitively associated with increased mortality, underscoring the critical importance of glycaemic control in this population. These categorizations shed light on the diverse glycaemic states within our patient cohort and emphasize the need for targeted interventions to optimize diabetes management and mitigate associated risks. In our cohort, 4.78% of the patients had HbA1c >10%.

Unfortunately, it is difficult to achieve target HbA1c values in HD patients. There are many factors underlying this situation. Patients often have poor compliance with a diabetic diet. These patients sometimes skip their oral antidiabetic medications because they use too many medications. They do not want to use insulin for fear of experiencing hypoglycaemia. Also, patients do not go for regular diabetes follow-up. However, despite all these negatives, the dialysis physician should not give up on this issue and should try to motivate the patient in this regard.

In HD patients, several factors can contribute to variability in HbA1c measurements, including anaemia, ESAs, iron, transfusions and acidosis. In CKD, inflammation, oxidative stress and metabolic acidosis can elevate HbA1c levels. Despite the findings of our study, with no significant correlation between haemoglobin levels, ESA dosage and HbA1c levels, it is crucial to acknowledge that the reliability of HbA1c measurements in reflecting blood sugar levels in HD is low [10]. Moreover, HbA1c generally does not provide information about glycaemic fluctuations and hypoglycaemic episodes. Alternative markers such as glycosylated albumin and fructosamine are available for long-term glycaemic control in advanced CKD, but their superiority remains debated. Notably, HbA1c measurements are relatively more cost-effective and accessible, leading the KDIGO guidelines to recommend HbA1c measurement [11].

In assessing glycaemic control among diabetic patients undergoing dialysis, a more rational approach involves utilizing at-home glucose monitoring and continuous subcutaneous glucose monitoring sensors. It is crucial to recognize the importance of reducing glycaemic fluctuations, not solely relying on HbA1c values, as this aligns with treatment goals [12]. In our study, 89.8% of patients used a glucometer and 75.82% engaged in at-home glucose monitoring. Notably, we observed a tendency for higher HbA1c levels in those experiencing symptomatic hypoglycaemia.

The responsibility for glycaemic control and its monitoring in HD patients prompts questions about who should manage these tasks. Our findings revealed that most patients received irregular care, often from any physician, and ≈8% managed their glycaemic control independently. While patients mainly sought assistance from dialysis or internal medicine physicians, referrals to endocrinology and nephrology specialists were notably low. This trend may be attributed to challenges in accessing an endocrinology specialist and the perception that other specialties (internal medicine or endocrinology) already oversee this aspect. However, we suggest that nephrologists, as primary medical caregivers of HD patients, should take a more proactive role, participate in monitoring and management and collaborate with HD physicians. By doing so, nephrology specialists can organize treatments and, when necessary, facilitate referrals to an endocrinologist, potentially yielding better outcomes. The observation that 4% of patients with elevated HbA1c levels are not receiving any antidiabetic treatment further underscores the importance of addressing this issue systematically and ensuring appropriate interventions for optimal patient care.

In our study, the average BMI of patients was 27.6 ± 5.5. When assessing the relationship between BMI and HbA1c, a higher BMI tended to be associated with elevated HbA1c levels. While obesity has traditionally been associated with increased mortality in diabetic patients, this relationship may be reversed in HD patients. This phenomenon, known as reverse epidemiology, suggests that an increasing BMI may have a protective effect on mortality. A meta-analysis including 81 423 patients supported this, revealing lower mortality rates in those with a BMI ≥25. The stability of haemodynamic status, cytokines and neurohormonal changes in patients with a higher BMI may contribute to this relationship [13, 14].

Additionally, in our study, an increase in the duration of diabetes correlated with higher HbA1c levels. Surprisingly, we found that patients adhering to a diabetic diet tended to have higher HbA1c levels. This finding suggests that patients who are recommended a professional diabetic diet are likely to be those with more uncontrolled diabetes, and it implies that referrals for dietary guidance may be overlooked in patients with relatively well-controlled HbA1c values. The lack of referrals to professional dieticians in patients with acceptable HbA1c levels could be the subject of further investigation of long-term outcomes. This observation also suggests that the diabetic diet in HD patients may be aimed at preventing hypoglycaemia rather than providing tight glycaemic control.

Given that diabetic patients often present with comorbid conditions such as hypertension, managing diabetes becomes more complex due to the interplay of chronic diseases and medications. Conflicting data exist on the relationship between comorbidities and HbA1c. Some studies show no clear relationship between the total number of comorbidities and HbA1c. One study with reverse results also considers the total number of comorbidities and HbA1c levels. This underscores the intricate nature of diabetes management in the presence of multiple health conditions [15, 16]. In our study, the group with higher HbA1c levels exhibited a higher prevalence of hypertension (84.8%), accompanied by poorer BP regulation. Additionally, these patients had higher UF volume, suggesting a potential link to non-compliance with dietary recommendations and hypervolaemia. Notably, this group also had higher rates of other comorbid conditions, such as CAD and low-output heart failure, while the rate of malignancy was lower. These findings align with the existing literature and highlight the intricate relationship between glycaemic control, cardiovascular health and other comorbidities.

Diabetic retinopathy, a significant microvascular complication often accompanying nephropathy, remains a critical consideration even after the development of diabetic nephropathy and ESKD. Vision is a critical motivating factor for diabetic dialysis patients. In our study, 63% of patients experienced decreased vision, which significantly impaired their quality of life. Glycaemic control remains essential, as hyperglycaemia can contribute to the onset or progression of retinopathy. A study of 1255 HD patients with type 2 diabetes found a significant association between glycaemic control and the presence of retinopathy [17]. Our study similarly demonstrated that higher HbA1c levels are linked to a greater prevalence of retinopathy, underscoring the crucial role of glycaemic control in dialysis patients, particularly in preventing diabetic retinopathy and associated visual impairment.

The existing literature also highlights a strong association between kidney disease, peripheral symmetric neuropathy, peripheral vascular disease, foot ulcers, amputation and survival in diabetic patients, suggesting that the risk increases shortly after the initiation of renal replacement therapy [18]. It has been reported that 6% of diabetic patients undergo amputation within the first 12 months of starting dialysis, compared with only 1% of non-diabetic patients. Several factors contribute to the association between dialysis and foot amputation in diabetes, including the effects of other diabetes-related complications (e.g. poor vision, peripheral neuropathy and peripheral arterial disease), although the precipitating factor is typically traumatic in nature. However, there is limited literature concerning HD patients, and our study contributes to filling this gap. In our dataset, the rate of lower extremity amputation was 7.5%, which exhibited an upward trend with higher HbA1c levels. This finding powerfully underscores the critical role of glycaemic control in HD patients, particularly in mitigating the risk of lower extremity amputations. It highlights the significance of targeted interventions and close monitoring to optimize outcomes in this patient population.

Hypoglycaemia is a common problem in diabetic HD patients. Conditions associated with an increased risk of hypoglycaemia in these patients include reduced gluconeogenesis in the kidneys, inadequate nutrition, decreased insulin clearance, glucose loss in the dialysis fluid, and glucose diffusion into erythrocytes during HD. HD-related hypoglycaemia is common during treatments with glucose-free dialysate, which creates a catabolic state similar to fasting [19]. HD-induced hypoglycaemia occurs more frequently in diabetic patients compared with non-diabetic patients. Therefore, insulin therapy and oral hypoglycaemic agents should be used with caution in dialysis patients. It underscores the importance of patient education and the necessity for personalized goals in managing diabetes in this population. In our study, we observed higher rates of symptomatic hypoglycaemia compared with the literature. However, upon reviewing the methodologies of those studies, it appears that the presence of hypoglycaemia (defined as a glucose level <70 mg/dl as measured in laboratory records) rather than symptomatic hypoglycaemia was used as the basis [19]. In our study, we reported the number of symptomatic hypoglycaemic events that required intervention. These numbers were obtained through patient interviews, as documenting symptomatic hypoglycaemia is very challenging. Therefore, further studies are needed to address this issue.

For diabetic HD patients, a critical step is to review and adjust diabetic medications at the initiation of HD [19]. As glomerular filtration rate decreases, dosage adjustments or contraindications may occur for some oral antidiabetic medications. Accumulation of active drug metabolites in plasma may increase the risk of prolonged hypoglycaemia, and insulin may be advantageous in this regard because of its short duration of action. All types of insulin can be used at low doses, considering the risk of hypoglycaemia and individual HbA1c targets [20, 21]. In our study, 71.5% of patients used insulin, with an intensive scheme being the preferred choice, especially in the group with poor glycaemic control. Mixed or long-acting insulins were also commonly chosen due to the convenience of use, with rates of 21.9% and 20.7%, respectively. Managing hypoglycaemia during HD requires careful monitoring, and the use of hyperglycaemic dialysate to prevent hypoglycaemia should be approached cautiously due to potential pro-inflammatory effects [21]. Reducing insulin doses during dialysis sessions, considering the absence of typical symptoms of hypoglycaemia and providing patients with snacks before meals are crucial considerations in optimizing glycaemic control and minimizing adverse events in this patient population. Among our patients, 21.7% were using OADs. When choosing OADs for diabetic HD patients, caution should be exercised, especially with sulfonylureas, as they carry a high risk of hypoglycaemia. Glycolazide, glimepiride and glipizide may be considered with close glucose control if necessary [20].

For stage 5 CKD patients, metformin and sodium–glucose co-transporter 2 inhibitors are not recommended due to potential risks [22]. Metformin use, in particular, has been debated regarding the risk of lactic acidosis. While some literature suggests that prolonged use of metformin in patients undergoing HD or peritoneal dialysis may not significantly increase the risk of lactic acidosis, risk factors to consider include not only renal functions, but also accompanying comorbidities such as infection and hypoxia [23]. In our patient population, 2% used metformin, likely to support insulin resistance and glycaemic control in specific cases.

Among dipeptidyl peptidase-4 (DPP-4) inhibitor drugs, linagliptin can be used without dose restriction in stage 5 CKD. The use of DPP-4 inhibitors in HD patients gradually increases, reaching ≈11% in our patients. These considerations underscore the complexity of medication management in diabetic HD patients and the importance of individualized treatment plans to optimize glycaemic control while minimizing risks.

There are some limitations to our study. First, it is retrospective. Although there are ≈60 000 HD patients in Turkey, only one-quarter of them could be reached. Second, other laboratory data, including HbA1c, are based on a single measurement. Third, histories of symptomatic attacks and emergency room visits were recorded based on patient statements. Despite these limitations, our study is the only study conducted in Turkey to date that provides information on glycaemic control and complications of a large number of diabetic HD patients.

In conclusion, our study highlights that the current treatments and target values for diabetic HD patients in our country show partial alignment with the 2022 KDIGO diabetes management guidelines. However, more efforts are needed to improve patient outcomes. Diabetic dialysis patients should undergo a comprehensive review and continuous monitoring by nephrologists and dialysis physicians. Establishing a multidisciplinary patient follow-up, including timely referrals to endocrinologists when necessary, is crucial to ensure optimal management. By implementing these measures, we can strive towards improving the quality of care and outcomes for diabetic HD patients, ultimately contributing to better overall patient well-being and survival rates.

## Data Availability

The data underlying this article will be shared upon reasonable request to the corresponding author.
